# Autophagosome protects proximal tubular cells from aldosterone-induced senescence through improving oxidative stress

**DOI:** 10.1080/0886022X.2021.1902821

**Published:** 2021-03-24

**Authors:** Jing-Yuan Cao, Li-lu Ling, Wei-Jie Ni, Hong-Lei Guo, Min Yang

**Affiliations:** a Department of Nephrology, Taizhou People’s Hospital, The Fifth Affiliated Hospital of Nantong University, Taizhou, China; b Department of Nephrology, Changzheng Hospital, Second Military Medical University, Shanghai, China; c Institute of Nephrology, Zhong Da Hospital, Southeast University School of Medicine, Nanjing, China; d Department of Nephrology, The First Affiliated Hospital of Nanjing Medical University, Nanjing, China; e Department of Nephrology, The Third Affiliated Hospital of Soochow University, Changzhou, China

**Keywords:** Proximal tubular cells, aldosterone, autophagy, senescence, oxidative stress

## Abstract

Aldosterone exerts an enormous function on proximal tubular cells (PTC) senescence, which is a common pathomechanism contributing to renal dysfunction. Numerous studies have shown that oxidative stress is deeply involved in the pathophysiologic processes of chronic kidney diseases. The study aims to investigate whether autophagy could regulate the process of senescence through oxidative stress in PTC both *in vivo* and *ex vivo*. Our results suggested that aldosterone treatment increased the senescence and oxidative stress as evidenced by increased percent of SA-β-Gal positive cells, reactive oxygen species level, expression of NADPH oxidase 4 (NOX4) rather than NOX2, and the up-regulation of p21 in cultured PTC. Furthermore, the alternation of the expression of p62 and LC3-II/LC3-I demonstrated that aldosterone treatment remarkably influenced autophagic flux. NOX4 siRNA treatment or autophagy induction with rapamycin reduced the oxidative stress and senescence in aldosterone-induced PTC. On the contrary, inhibition of autophagy with chloroquine worsened these changes. Similar results were further confirmed *in vivo*. Our results suggested that autophagy may become a realistic therapeutic strategy against aldosterone-induced PTC injury *via* improving oxidative stress.

## Introduction

Cell senescence, which is a complex state characterized by cell cycle arrest, contributes to renal dysfunction [1,2]. Over the past few decades, plenty of studies have documented that proximal tubular cells (PTC) senescence is deeply involved in the pathogenesis of kidney diseases, including diabetic nephropathy [3], renal aging [4], chronic CsA nephropathy [5], and so on. Aldosterone (Aldo), which is considered as a significant risk factor of chronic kidney disease (CKD), has become a focus of research [6]. Recently, studies indicated that cyclin-dependent kinase inhibitors, p21 and p53, were likely to participate in Aldo-induced PTC senescence, and this kind of aging was different from replicating aging [7]. Inhibiting the process of senescence could be an effective strategy to prevent the progression of kidney diseases.

A great deal of evidence showed that oxidative stress (OS) exerts an enormous influence on the senescence of PTC [2]. OS is a complex process featured by abnormal reactive oxygen species (ROS) accumulation, which is deemed to be the reason for DNA oxidative damages and mitochondrial dysfunction [8]. Previous studies showed that ROS is released mainly from damaged mitochondrial and NADPH oxidase (NOX), including NOX1, NOX2, NOX4, et al. [9]. Our previous research demonstrated that ROS production pushed forward an immense influence on the pathogenesis of Aldo-induced kidney injury [10,11]. Therefore, attenuating ROS production is considered as a novel therapeutic strategy on Aldo-induced tubular senescence.

Autophagy is an essential, conserved self-eating process that cells perform to allow the degradation of intracellular components. The process requires the formation of a double-membrane structure containing the sequestered cytoplasmic material, the autophagosome, which ultimately fuses with the lysosome [12]. Plenty of studies have demonstrated that autophagy rises a key function in the regulation of aging process [13,14]. Nevertheless, it is not clear whether autophagy is related to Aldo-induced senescence in PTC. Hence, we hypothesize that autophagy may protect PTC from Aldo-induced senescence both *in vivo* and *in vitro*. This study aims at verifying the effects of the autophagy process on senescence of Aldo-induced PTC and exploring the possible molecular mechanism related to OS. Our results indicated the protective role of autophagy against Aldo-induced PTC senescence through down-regulation of OS, a mechanism that could be the regulating target to reduce the Aldo-induced renal injury.

## Materials and methods

### Materials

Aldo, Rapamycin (Rap), and Chloroquine (CQ) were obtained from Sigma Aldrich (St. Louis, MO). Anti-LC3 (4108) and anti-GAPDH (8884) antibodies were purchased from Cell Signaling Technology (Beverly, MA). Anti-p62 (ab109012), Anti-p21 (ab109199), anti-NOX2 (ab129068) and anti-NOX4 (ab109225) antibodies were purchased from Abcam (Cambridge, MA). Anti-β-actin (AB2001) was purchased from Abways. ROS/RNS Assay Kit (ab238535) was purchased from Abcam (Cambridge, MA).

### Animal preparation

Animal models were prepared as previously reported [15]. All experimental procedures were performed according to the guidelines for the care and use of animals established by Nantong University. Twenty-four healthy male rats (Sprague-Dawley, 180–200 g) were housed under standard conditions and randomly assigned to 4 groups (*n* = 6) as follows: control group, Aldo group, Aldo + Rap group, and Aldo + CQ group. All of the rats underwent a right uninephrectomy operation in the first week to generate a disease model. Two weeks later, osmotic minipump (Alzet, Cupertino, CA; model 2004) was used to deliver Aldo (0.75 μg/h) to rats in Aldo group, Aldo + Rap group and Aldo + CQ group, or vehicle in control group. The delivery of Rap (1 mg/kg per day, intraperitoneal [ip]) or CQ (60 mg/kg per day, ip) was synchronized with Aldo. The rats in control group and Aldo group were treated with isopynic solvent. Rap or CQ were dissolved in DMSO (0.5%) and phosphate buffer saline (PBS, 99.5%). During the experimental period, the drinking water of all groups was supplemented with 1% NaCl. Blood and kidney tissues were harvested after 4 weeks. Partial kidney tissues were quickly removed and stored in a freezing tube at −80 °C. The remaining renal samples were fixed in paraformaldehyde for histological examination.

### Cell culture

The human kidney epithelial cell line HK-2 was obtained from ATCC and cultured in DMEM/F12 medium supplemented with 10% fetal bovine serum (FBS). HK-2 cells were incubated with the following four drug combinations: no drug control, Aldo (100 nM, 24 h), Aldo + Rap (1 mM, 24 h), Aldo + CQ (20 μM, 24 h). Rap and CQ were dissolved in DMSO. DMSO was used as a control.

To knockdown NOX4 in HK-2 cells, 80 nM siRNA specifically targeting NOX4 or negative control (Con) siRNA was transfected using Lipofectamine 3000 (Invitrogen, Carlsbad, CA) according to the manufacturer’s instructions. Nox4 siRNA sequence is as follow: GCAGGAGAACCAGGAGAUU'.

### Senescence-associated beta-galactosidase (SA-β-Gal) staining

Senescence-associated SA-β-gal staining was performed as described in the manufacturer's protocol (Cell Signaling Technology).

HK-2 cells seeded in six-well plates were treated as described previously. After 24 h, cells were washed with PBS and fixed in 2% formaldehyde/0.2% glutaraldehyde. Then the fixed cells were incubated overnight at 37 °C (without CO_2_) with SA-β-Gal staining solution. Subsequently, we chose 100 to 200 cells randomly in six microscopic fields and counted the positive cells to determine the percentages.

The cryostat sections of kidney tissues were incubated with SA-β-Gal staining solution at 37 °C overnight. To maintain its sensitivity, we performed the staining at pH 5.5 at all times. To demonstrate morphology, we stained tissues with eosin after SA-β-Gal staining. SA-β-Gal-positive cells were counted in five randomly chosen fields per section at ×100 magnification stained in blue.

### ROS production

Relative changes of ROS levels in HK-2 cells were detected by 2′, 7′-dichlo-rofluorescein (DCF) at 37 °C in the dark using ROS/RNS Assay Kit following the instructions. Then cells were washed twice in PBS, and fluorescence was measured using a fluorescent microplate reader (FlexStation II384, MolecularDevice, CA).

### Immunofluorescence staining

The HK-2 cells were fixed with cold acetone. 3% bovine serum albumin (BSA) was used to block antigen and the cells were incubated with the primary antibody against LC3 (1:200) at 4 °C overnight. Then, we washed the sections with PBS and incubated them with a tetramethylrhodamine isothiocyanate (TRITC)-conjugated secondary antibody (Sigma-Aldrich) in the dark for 1 h. After being washed with PBS, cells were visualized with a Nikon Eclipse 80i Epi-fluorescence microscope.

### Dihydroethidium (DHE) staining

Frozen kidney segments were stained with DHE (50 μM, Invitrogen, Carlsbad, CA) in the dark (37 °C, 30 min). Laserscanning confocal microscope system (Bio-Rad Laboratories, Hercules, CA) was used in Image acquisition to assess the intensity. The intensities of DHE fluorescence in the renal tubules were assessed and calculated with Image pro plus 6.0 software.

### Immunohistochemistry

Immunohistochemical stains were performed using 3-μm thick renal sections. After antigen retrieval, the sections were incubated with the primary antibodies against LC3 (1:1000) and NOX4(1:200), then HRP-coupled secondary antibodies (Vectastain elite, Vector Labs) were used to label them. All experiments were repeated three times.

### Electron microscopy

Kidney tissues were first fixed in a solution containing 2.5% formaldehyde-glutaraldehyde, then postfixed in a 1% osmium tetroxide solution and dehydrated in ethanol. The propylene oxide was used to replace the ethanol in the tissues. Finally, we embedded the blocks with an epoxy resin and sliced them into ultrathin sections. The sections were double-stained with lead citrate and uranyl acetate. All the sections were observed by transmission electron microscope (TEM, JEOL JEM-1010, Tokyo, Japan).

### Western blotting

Proteins from kidney tissues or HK-2 cells were separated by SDS-PAGE and transferred to a nitrocellulose membrane. Then we incubated the membranes with the primary antibodies against LC3 (1: 1000), P62 (1:1000), NOX2 (1:1000), NOX4 (1:1000), p21 (1:1000), or GAPDH (1:1000) at 4 °C overnight. After three washes with PBS, we incubated the nitrocellulose membranes with HRP-conjugated secondary antibodies for 1 h at room temperature. Finally, the Amersham ECL Detection System (Amersham, Buckinghamshire, UK) was used to visualize the membranes. Quantity One Software (Bio-Rad) was adopted to quantify the densitometry of immunoreactive bands.

## Statistical analyses

All data were analyzed using SPSS 19.0 statistics software. Comparisons between groups were using one-way ANOVA followed by Dunnett’s multiple comparison tests or Student’s t-test. *p* < 0.05 was considered statistically significant.

## Results

### Effect of NOX4 in Aldo-induced OS and senescence in HK-2 cells

**Figure 1.**
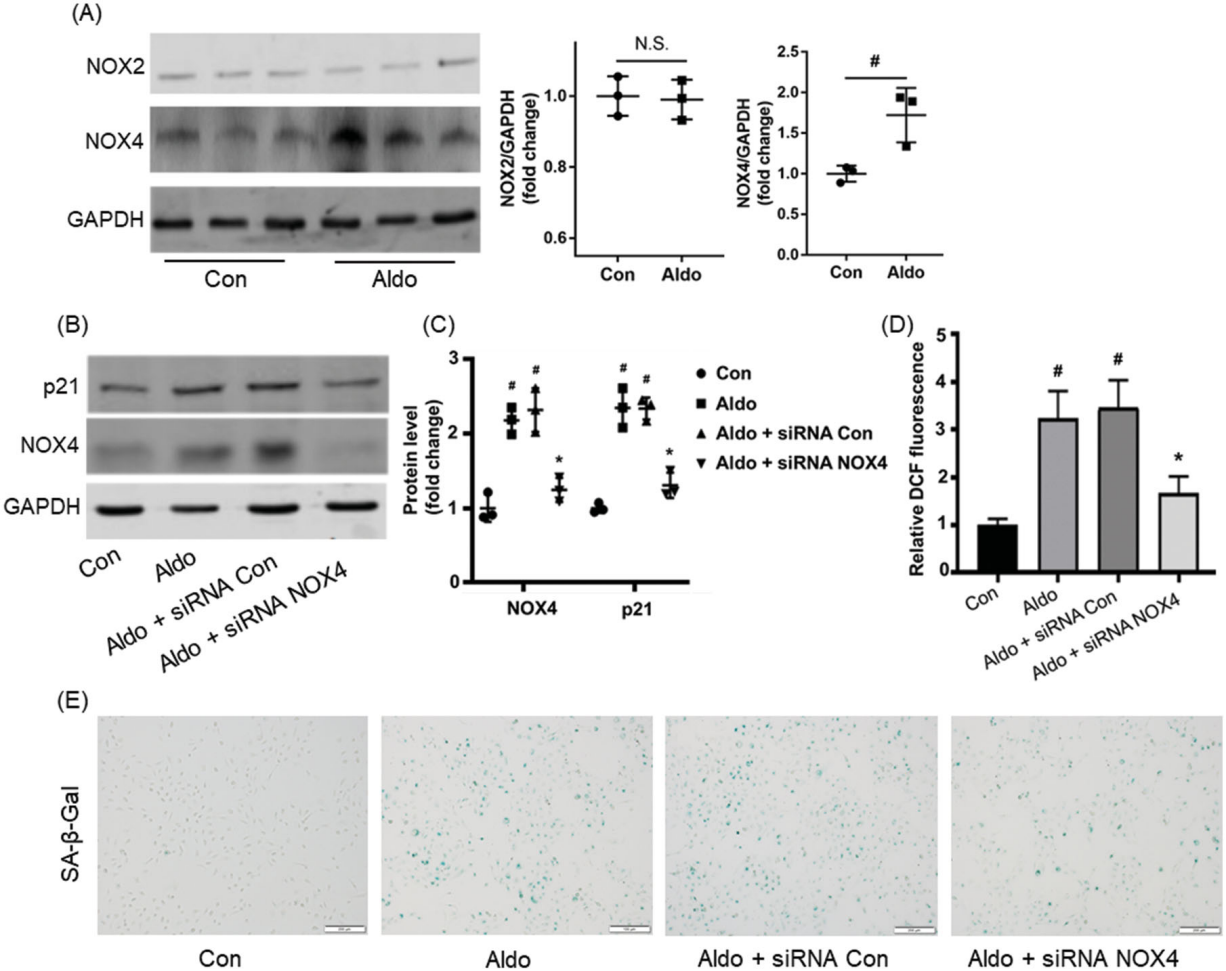
NOX4 is mediated at Aldo-induced OS and senescence in HK-2 cells. (A) Western blot analysis showed the expression of NOX4, NOX2, and GAPDH proteins in HK-2 cells after treatment without or with 100nM Aldo for 24 h (*n* = 3). (B) Equal numbers of HK-2 cells were incubated in media containing buffer (Control), siRNA-Con or siRNA-NOX4 with or without Aldo for 24 h as indicated. The whole cell lysate was immunoblotted with antibodies against NOX4, p21, and GAPDH (*n* = 3). (C) The graphical presentation showed the relative abundance levels of NOX4 and p21 after normalization with GAPDH (*n* = 3). (D) Quantification of 2’, 7’-dichlo-rofluorescein (DCF) fluorescence in various groups as indicated (*n* = 3). (E) SA-β-gal activity, which appears as bright-blue granular staining in the cytoplasm of HK-2 cells in various groups as indicated (*n* = 3). ^#^*p* < 0.05 vs. normal control, *p < 0.05 vs. Aldo alone or Aldo + siRNA Con.

Because OS contributed to Aldo-induced PTC injury and NADPH oxidases were known as a source of ROS, we firstly detected the level of NOX2 and NOX4 (the two main members of NADPH oxidases family). Interestingly, Aldo treatment increased the expression of NOX4, but not NOX2 (Figure 1(A)). NOX4 siRNA intervention abrogated the expression of p21 (Figure 1(B,C)), ROS level (Figure 1(D)) and the SA-β-Gal staining positive senescence cells (Figure 1(E)) induced by Aldo, suggesting that NOX4-mediated OS was partially responsible for Aldo-induced cell senescence.

### Inhibition of autophagy aggravated Aldo-induced OS and senescence in HK-2 cells

**Figure 2.**
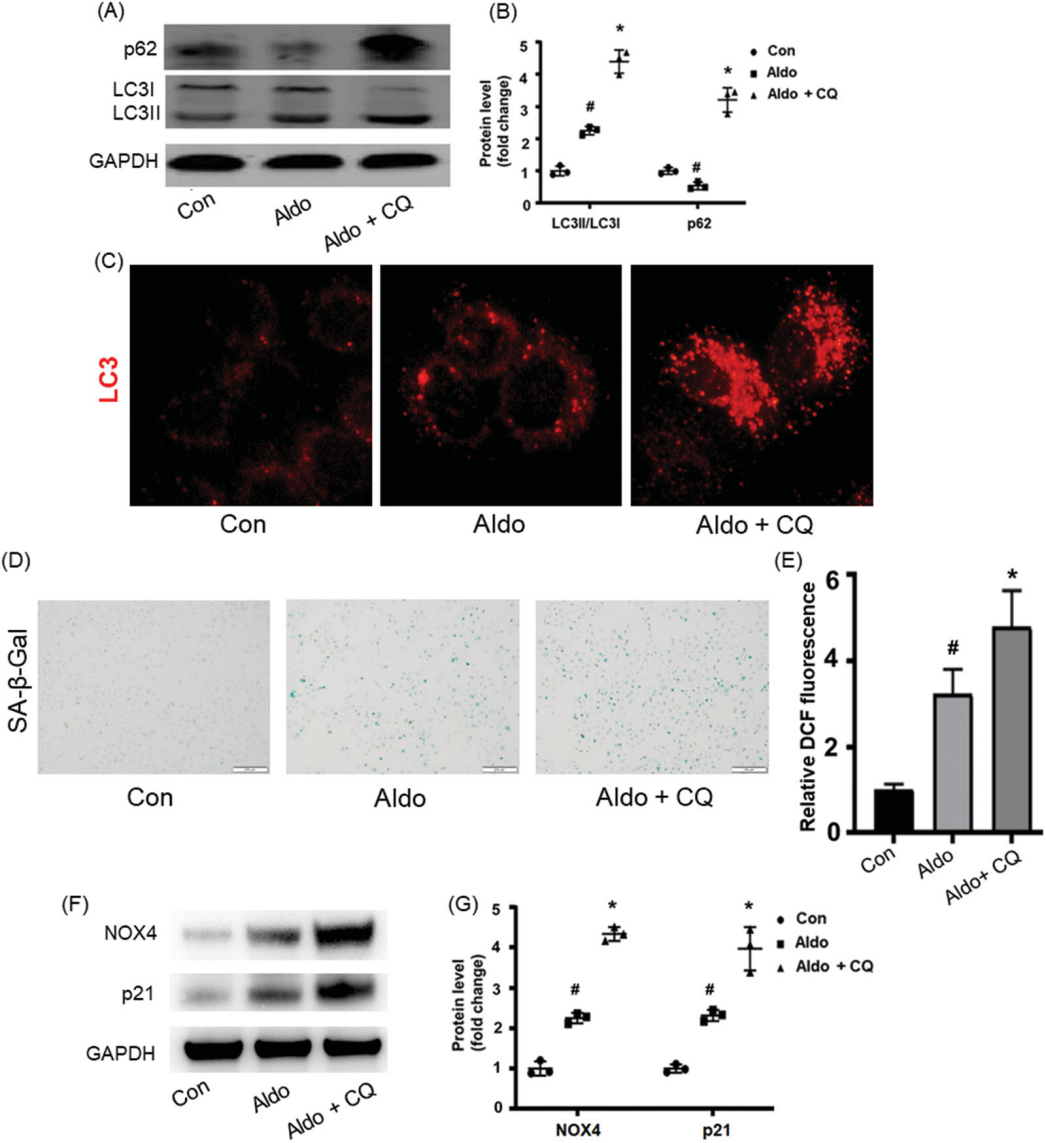
Blocking autophagy worsened Aldo-induced OS and senescence in HK-2 cells. (A) Western blot analysis revealed the expression of LC3-II/LC3-I, p62, and GAPDH proteins in HK-2 cells after various treatments as indicated (*n* = 3). (B) The graphical presentation indicates the relative abundance levels of LC3-II/LC3-I and p62 after normalization with GAPDH (*n* = 3). (C) Immunofluorescence staining for LC3 in HK-2 cells, which were incubated in media containing buffer (Control), Aldo, or Aldo + CQ for 24 h as indicated (n = 3). (D) SA-β-gal activity, which appears as a bright-blue granular staining in the cytoplasm of HK-2 cells in various groups as indicated (*n* = 3). (E) Quantification of 2’, 7’-dichlo-rofluorescein (DCF) fluorescence in various groups as indicated (n = 3). (F) Western blot analysis showed the expression of p21 and GAPDH proteins in HK-2 cells after various treatments as indicated (*n* = 3). (G) Graphical presentation shows the relative abundance levels of p21 and NOX4 after normalization with GAPDH (*n* = 3). ^#^*p* < 0.05 vs. normal control, *p < 0.05 vs. Aldo alone.

As CQ can inhibit autophagic flux by decreasing the formation of autolysosome [16], we detected the level of p62 and LC3-II/LC3-I in the presence or absence of CQ in Aldo-treated HK-2 cells to investigate whether impaired autophagic flux could affect OS and senescence. The results showed that Aldo induced a striking alternation of LC3-II/LC3-I ratio and the expression of p62, both of which were enhanced after CQ treatment (Figure 2(A,B)). Immunofluorescence staining of LC3 confirmed these results (Figure 2(C)). All these data indicated that Aldo treatment increased autophagy flux in HK-2 cells *in vitro*.

To further prove the impact of autophagy in Aldo-induced senescence, we evaluated the senescence parameters in the presence of CQ. Compared with the Aldo group, it was found that CQ treatment significantly increased the SA-β-Gal positive staining rate (Figure 2(D)), ROS level (Figure 2(E)), and the expressions of p21 (Figure 2(F,G)). These results demonstrated that inhibiting autophagy worsened Aldo-induced PTC senescence *in vitro*.

### Activating autophagy improved Aldo-induced OS and senescence in HK-2 cells

**Figure 3.**
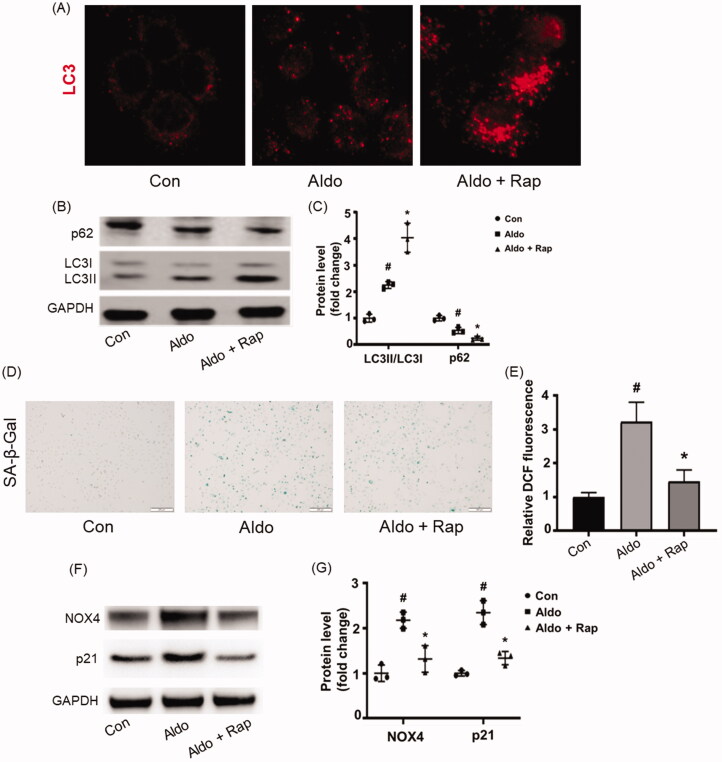
Rap improved Aldo-induced OS and senescence in HK-2 cells. (A) Immunofluorescence staining for LC3 in HK-2 cells, which were incubated in media containing buffer (Control) or Aldo with or without Rap for 24 h as indicated (*n* = 3). (B) Western blot analysis revealed the expression of LC3-II/LC3-I, p62, and GAPDH proteins in HK-2 cells after various treatments as indicated. (C) Graphical presentation indicated the relative abundance levels of LC3-II/LC3-I and p62 after normalization with GAPDH (*n* = 3). (D) SA-β-gal activity, which appears as bright-blue granular staining in the cytoplasm of HK-2 cells in various groups as indicated (*n* = 3). (E) Quantification of 2’, 7’-dichlo-rofluorescein (DCF) fluorescence in various groups as indicated (*n* = 3). (F) Western blot analysis showed the expression of p21, NOX4, and GAPDH proteins in HK-2 cells after various treatments as indicated (*n* = 3). (G) Graphical presentation shows the relative abundance levels of p21 and NOX4 after normalization with GAPDH (*n* = 3). ^#^*p* < 0.05 vs. normal control, *p < 0.05 vs. Aldo alone.

Rap is the most common strategy of autophagy activation [17]. To investigate the impact of autophagy in Aldo-induced PTC senescence, Rap was applied to treat the HK-2 cells. Compared to cells treated with Aldo alone, Rap further increased LC3 dots (Figure 3(A)), LC3-II/LC3-I ratio and decreased the expression of p62 (Figure 3(B,C)), suggesting that Rap treatment further enhanced the autophagy in HK-2 cells, with remarkably reduced Aldo-induced OS and senescence shown in Figure 3(D,E). Furthermore, lower expression of p21 was detected after Rap treatment compared with Aldo groups (Figure 3(F,G)).

### *Aldo induced autophagy in kidney proximal tubules* in vivo

**Figure 4.**
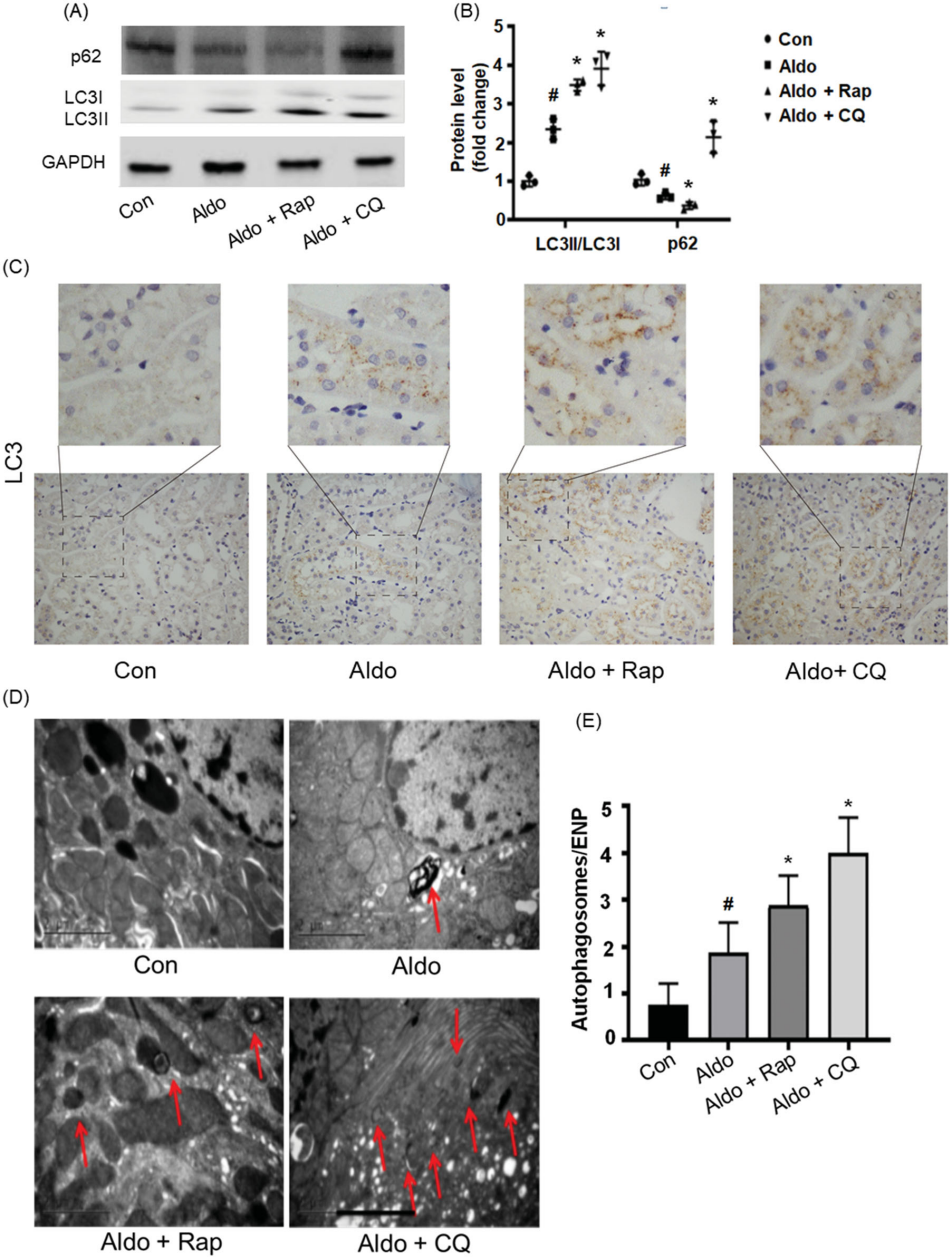
Aldo induced autophagy in kidney proximal tubules *in vivo*. (A) Western blot analysis revealed the expression of LC3-II/LC3-I, p62, and GAPDH proteins after various treatments in rats, as indicated (*n* = 3). (B) Graphical presentation showed the relative abundance levels of LC3-II/LC3-I and p62 after normalization with GAPDH (*n* = 3). (C) Immunohistochemical staining for LC3 in rat kidney tissue from various groups, as indicated (*n* = 6). (D) Representative electron micrographs showing autophagic vacuoles in PTCs (*n* = 6). The red arrow indicates autophagosomes. Scale bar = 2 μm. (E) Bar graph indicating the Autophagosomes/ENP in various groups (*n* = 6). ^#^*p* < 0.05 vs. normal control, *p < 0.05 vs. Aldo alone.

In order to further explore the role of autophagy in PTC senescence, a rat model induced by Aldo was established. As shown in Supplementary Figure 1, Aldo significantly increased the levels of serum creatinine (SCr) and blood urea nitrogen (BUN), while the treatment of Rap attenuated the kidney injury. In contrast, CQ treatment worsened the Aldo-induced damage (Supplementary Figure 1). Figure 4(A) showed that Aldo significantly up-regulated the ratio of LC3-II/LC3-I in renal tissues and enhanced degradation of p62 (Figure 4(A,B)). Immunohistochemical staining of LC3 showed the formation of autophagosomes in kidneys. Consistent with the above results, LC3 was diffusely distributed throughout the cells, while LC3 was punctated in control group. Furthermore, intense dot-like LC3 staining puncta appeared in Aldo treated group indicating the formation of autophagosomes (Figure 4(C)). To further detect the autophagy flux in proximal tubule cells, electron microscopy was used to evaluate the formation of autophagic vacuoles (Figure 4(D,E)). We explored the levels of mineralocorticoid receptor at the same time. It showed that Rap or CQ did not change the expression of mineralocorticoid receptor (Supplementary Figure 2). In brief, these results provided convincing proof for the occurrence of autophagy in Aldo-induced renal damage. As expected, treatment with Rap increased autophagic activity, whereas CQ treatment blocked autophagy in Aldo-infused rats (Figure 4).

### *Autophagy regulated Aldo-induced senescence and oxidative stress in PTC* in vivo

**Figure 5.**
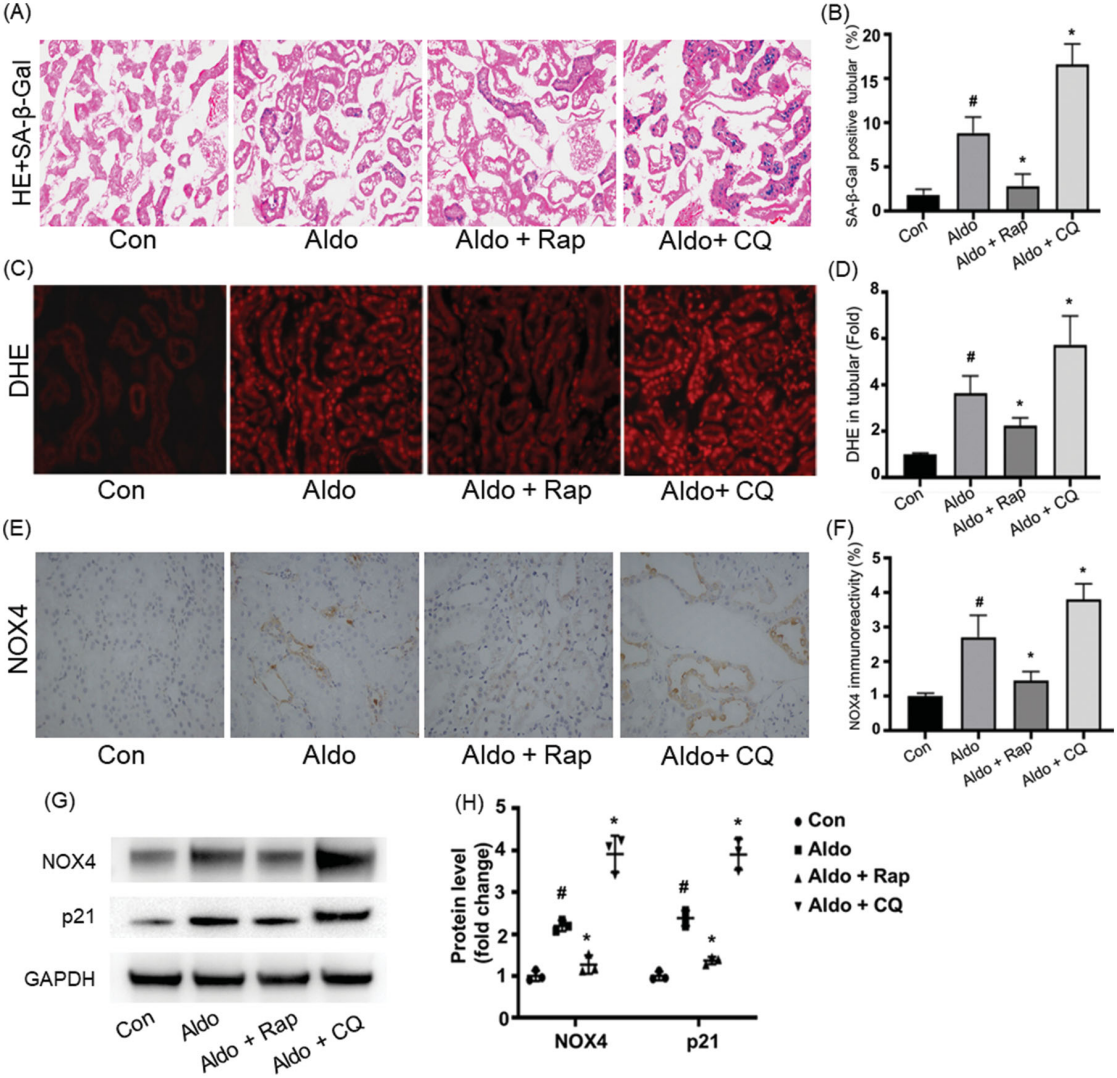
Autophagy regulated OS and senescence in PTCs of Aldo-induced rats. (A) Senescence-associated-galactosidase (SA-β-Gal) Staining in rat renal cortex (*n* = 6). SA-β-Gal is labeled by bright-blue in the PTCs. (B) Bar graph indicating the percentage of SA-β-Gal positive cells per field in tubular. (C) DHE staining of rat kidney sections (*n* = 6). (D) Bar graph indicating the mean DHE intensity per field in rat tubular cells. (E) Immunohistochemical staining for NOX4 in rat kidney tissues from various groups, as indicated (*n* = 6). (F) Bar graph indicating NOX4 immunoreactivity per field in rat tubular cells. (G) Western blot analysis revealed the expression of NOX4, p21, and GAPDH proteins after various treatments in rats (*n* = 3). (H) Graphical presentation shows the relative abundance levels of NOX4 and p21 after normalization with GAPDH (*n* = 3). ^#^*p* < 0.05 vs. normal control, *p < 0.05 vs. Aldo alone.

In accordance with a previous study [18], infusion with Aldo increased the SA-β-Gal staining in PTC of rats, but not in vehicle-infused rats (Figure 5(A)). Activation of autophagy by Rap reduced the SA-β-Gal activity, while the inhibition of autophagy by CQ worsened the Aldo-induced SA-β-Gal activity changes in PTC of rats. To prove autophagy could protect PTC against senescence *via* reducing OS in Aldo-infused rats, we detect the DHE level to evaluate the OS status in rats from each group. Compared with the control rats, the DHE level was elevated in PTC of Aldo-induced rats (Figure 5(C,D)). Similar to the *in vitro* study, Aldo infusion increased the expression of NOX4 (Figure 5(E,F)). Rap treatment abrogated the up-regulation of DHE and decreased the expression of NOX4. As shown in Figure 5(G,H), Aldo infusion remarkably up-regulated the level of p21. On the contrary, CQ treatment further enhanced OS and the expression of NOX4 in PTC in Aldo-induced rats (Figure 5). Taken together, these results suggested that autophagy played a protective role against the senescence of PTC in this experimental animal model.

## Discussion

In this study, we demonstrated that NOX4 mediated OS and senescence in Aldo-induced kidney injury. Meanwhile, autophagy could attenuate Aldo-induced PTC senescence through improving OS. Thus, it is considered that regulating autophagy process may be in favor of attenuating Aldo-induced PTC injury.

Growing evidence demonstrates that the activation of renin-angiotensin-aldosterone system (RAAS) is the typical feature of CKD [19,20]. Interruption of the RAAS, *via* angiotensin-converting enzyme (ACE) inhibitors and angiotensin receptor blockers (ARBs), remarkably reduces the morbidity and mortality in patients with CKD [21,22]. However, the phenomenon of Aldo breakthrough signifies incomplete blockade of RAAS activation [23]. Moreover, a great deal of evidence suggests that Aldo concentration is an independent risk factor for CKD. Hence, Aldo has become a hot spot for CKD in recent years [24].

In line with a previous study [18], our research demonstrated that Aldo significantly up-regulated the production of ROS in podocytes [11]. Brem et al. pointed that Aldo-induced ROS production may be the main source for uncontrolled OS [25]. Growing evidence has proved that Aldo-induced cell injury is extensively involved in renal disease [14]. As a result, we should attach great importance to the OS induced by Aldo in PTC.

Previous studies have suggested that renal cortical ROS levels are related to Aldo/salt-induced kidney injury [26]. Infusion of Aldo contributed to senescence, apoptosis, and tubulointerstitial fibrosis both *in vivo* and *in vitro* [27]. Furthermore, cells would move toward senescence, dedifferentiate into other phenotypes, and ultimately undergo apoptosis when excessive stress cannot be managed by the endogenous repair system [2,28–30]. Thus, improving senescence at the early stage of Aldo-induced PTC injury is a potential intervene target. As far as we know, OS, which is featured by the increased generation of ROS and DNA oxidative damage, participates in the Aldo-induced renal injury [31,32]. Nevertheless, the source of ROS is still under investigation in Aldo-induced PTC injury model. Our results showed that increased NOX4 rather than NOX2 is responsible for the increase of ROS in Aldo-induced PTC senescence.

Autophagy has been proved to play beneficial effects against senescence [14]. Impaired autophagy can result in overt OS while increased autophagy can improve the damage of overt OS [33–35]. However, increased OS was not the reason for impaired autophagic flux. On the contrary, Aldo increased autophagic flux. Our research answered the question that whether autophagy can regulate the Aldo-induced senescence through regulating OS. The process of autophagy contains the formation of autophagosome and fuses with the lysosome [12]. LC3-II/LC3-I and p62 were used to determine the condition of autophagy flow. As expected, enhanced autophagy significantly inhibited Aldo-induced OS and senescence. Meanwhile, inhibiting autophagy worsened these changes. Autophagy is an evolutionarily conserved catabolic process, which can remove damaged cellular components and maintain energy homeostasis [36], including the damaged mitochondrial (another source of ROS). It is not strange that regulating autophagy process can anti-OS but has no effect on the level of NOX2 in Aldo-induced PTC injury.

In this study, we found that autophagy was deeply involved in the pathomechanism of Aldo-induced PTC injury. Furthermore, regulating OS through autophagy may become a good target for therapeutic strategies of CKD.

## Supplementary Material

Supplemental Material

## References

[1] He S, Sharpless NE. Senescence in health and disease. Cell. 2017;169(6):1000–1011. 28575665 10.1016/j.cell.2017.05.015 PMC5643029

[2] Schmitt R, Melk A. Molecular mechanisms of renal aging. Kidney Int. 2017;92(3):569–579. 28729036 10.1016/j.kint.2017.02.036

[3] Xiong Y, Zhou L. The signaling of cellular senescence in diabetic nephropathy. Oxid Med Cell Longev. 2019;2019:7495629. 31687085 10.1155/2019/7495629 PMC6794967

[4] Gekle M. Kidney and aging - a narrative review. Exp Gerontol. 2017;87(Pt B):153–155. 27032877 10.1016/j.exger.2016.03.013

[5] Jennings P, Koppelstaetter C, Aydin S, et al. Cyclosporine A induces senescence in renal tubular epithelial cells. Am J Physiol Renal Physiol. 2007;293(3):F831–838. 17596534 10.1152/ajprenal.00005.2007

[6] Briet M, Schiffrin EL. Aldosterone: effects on the kidney and cardiovascular system. Nat Rev Nephrol. 2010;6(5):261–273. 20234356 10.1038/nrneph.2010.30

[7] Kim YY, Jee HJ, Um JH, et al. Cooperation between p21 and Akt is required for p53-dependent cellular senescence. Aging Cell. 2017;16(5):1094–1103. 28691365 10.1111/acel.12639 PMC5595696

[8] Bazopoulou D, Knoefler D, Zheng Y, et al. Developmental ROS individualizes organismal stress resistance and lifespan. Nature. 2019;576(7786):301–305. 31801997 10.1038/s41586-019-1814-y PMC7039399

[9] Zhang J, Wang X, Vikash V, et al. ROS and ROS-mediated cellular signaling. Oxid Med Cell Longev. 2016;2016:4350965. 26998193 10.1155/2016/4350965 PMC4779832

[10] Ding W, Guo H, Xu C, et al. Mitochondrial reactive oxygen species-mediated NLRP3 inflammasome activation contributes to aldosterone-induced renal tubular cells injury. Oncotarget. 2016;7(14):17479–17491. 27014913 10.18632/oncotarget.8243 PMC4951227

[11] Wang B, Xu X, He X, et al. Berberine improved aldo-induced podocyte injury via inhibiting oxidative stress and endoplasmic reticulum stress pathways both in vivo and in vitro. Cell Physiol Biochem. 2016;39(1):217–228. 27336740 10.1159/000445618

[12] Dikic I, Elazar Z. Mechanism and medical implications of mammalian autophagy. Nat Rev Mol Cell Biol. 2018;19(6):349–364. 29618831 10.1038/s41580-018-0003-4

[13] Rajendran P, Alzahrani AM, Hanieh HN, et al. Autophagy and senescence: a new insight in selected human diseases. J Cell Physiol. 2019;234(12):21485–21492. 31144309 10.1002/jcp.28895

[14] Rubinsztein DC, Mariño G, Kroemer G. Autophagy and aging. Cell. 2011;146(5):682–695. 21884931 10.1016/j.cell.2011.07.030

[15] Ding W, Xu C, Wang B, et al. Rotenone attenuates renal injury in aldosterone-infused rats by inhibiting oxidative stress, mitochondrial dysfunction, and inflammasome activation. Med Sci Monit. 2015;21:3136–3143. 26474533 10.12659/MSM.895945 PMC4614375

[16] Mauthe M, Orhon I, Rocchi C, et al. Chloroquine inhibits autophagic flux by decreasing autophagosome-lysosome fusion. Autophagy. 2018;14(8):1435–1455. 29940786 10.1080/15548627.2018.1474314 PMC6103682

[17] Babchia N, Calipel A, Mouriaux F, Faussat AM, et al. The PI3K/Akt and mTOR/P70S6K signaling pathways in human uveal melanoma cells: interaction with B-Raf/ERK. Invest Ophthalmol Vis Sci. 2010;51(1):421–429. 19661225 10.1167/iovs.09-3974

[18] Fan YY, Kohno M, Hitomi H, et al. Aldosterone/Mineralocorticoid receptor stimulation induces cellular senescence in the kidney. Endocrinology. 2011;152(2):680–688. 21190955 10.1210/en.2010-0829

[19] Nagase M, Fujita T. Role of Rac1-mineralocorticoid-receptor signalling in renal and cardiac disease. Nat Rev Nephrol. 2013;9(2):86–98. 23296296 10.1038/nrneph.2012.282

[20] Lerman LO. Imaging: BOLD assessment-effects of RAAS inhibition in CKD. Nat Rev Nephrol. 2014;10(5):247–248. 24662436 10.1038/nrneph.2014.58

[21] Zhang Y, He D, Zhang W, et al. ACE inhibitor benefit to kidney and cardiovascular outcomes for patients with non-dialysis chronic kidney disease stages 3–5: a network meta-analysis of randomised clinical trials. Drugs. 2020;80(8):797–811. 32333236 10.1007/s40265-020-01290-3 PMC7242277

[22] Baltatzi M, Savopoulos C, Hatzitolios A. Role of angiotensin converting enzyme inhibitors and angiotensin receptor blockers in hypertension of chronic kidney disease and renoprotection. Study results. Hippokratia. 2011;15(Suppl 1):27–32. 21897755 PMC3139675

[23] Staessen J, Lijnen P, Fagard R, et al. Rise in plasma concentration of aldosterone during long-term angiotensin II suppression. J Endocrinol. 1981;91(3):457–465. 7035596 10.1677/joe.0.0910457

[24] Hostetter TH, Ibrahim HN. Aldosterone in chronic kidney and cardiac disease. J Am Soc Nephrol. 2003;14(9):2395–2401. 12937319 10.1097/01.asn.0000086472.65806.73

[25] Brem AS, Gong R. Therapeutic targeting of aldosterone: a novel approach to the treatment of glomerular disease. Clin Sci (Lond). 2015;128(9):527–535. 25671776 10.1042/CS20140432 PMC4356246

[26] Nishiyama A, Kusaka T, Kitajima H. [Role of aldosterone in oxidative stress and renal injury]. Yakugaku Zasshi. 2007;127(9):1331–1337. 17827915 10.1248/yakushi.127.1331

[27] Wang B, Ding W, Zhang M, et al. Rapamycin attenuates aldosterone-induced tubulointerstitial inflammation and fibrosis. Cell Physiol Biochem. 2015;35(1):116–125. 25547416 10.1159/000369680

[28] Infante B, Franzin R, Madio D, et al. Molecular mechanisms of AKI in the elderly: from animal models to therapeutic intervention. JCM. 2020;9(8):2574. 10.3390/jcm9082574 PMC7464895 32784471

[29] Franzin R, Stasi A, Fiorentino M, et al. Inflammaging and complement system: a link between acute kidney injury and chronic graft damage. Front Immunol. 2020;11:734. 32457738 10.3389/fimmu.2020.00734 PMC7221190

[30] Castellano G, Franzin R, Sallustio F, et al. Complement component C5a induces aberrant epigenetic modifications in renal tubular epithelial cells accelerating senescence by Wnt4/βcatenin signaling after ischemia/reperfusion injury. Aging (Albany NY). 2019;11(13):4382–4406. 31284268 10.18632/aging.102059 PMC6660044

[31] Schieber M, Chandel NS. ROS function in redox signaling and oxidative stress. Curr Biol. 2014;24(10):R453–E462. 24845678 10.1016/j.cub.2014.03.034 PMC4055301

[32] Yang M, Wang B, Li M, et al. Connexin 43 is involved in aldosterone-induced podocyte injury. Cell Physiol Biochem. 2014;34(5):1652–1662. 25401388 10.1159/000366367

[33] Li L, Tan J, Miao Y, et al. ROS and autophagy: interactions and molecular regulatory mechanisms. Cell Mol Neurobiol. 2015;35(5):615–621. 25722131 10.1007/s10571-015-0166-x PMC11486209

[34] Filomeni G, De Zio D, Cecconi F. Oxidative stress and autophagy: the clash between damage and metabolic needs. Cell Death Differ. 2015;22(3):377–388. 25257172 10.1038/cdd.2014.150 PMC4326572

[35] Sureshbabu A, Ryter SW, Choi ME. Oxidative stress and autophagy: crucial modulators of kidney injury. Redox Biol. 2015;4:208–214. 25613291 10.1016/j.redox.2015.01.001 PMC4803795

[36] Galluzzi L, Baehrecke EH, Ballabio A, et al. Molecular definitions of autophagy and related processes. Embo J. 2017;36(13):1811–1836. 28596378 10.15252/embj.201796697 PMC5494474

