# Supplementary material for: Autophagosome protects proximal tubular cells from aldosterone-induced senescence through improving oxidative stress
Source: Ren Fail. 2021 Mar 24;43(1):556–65. doi: 10.1080/0886022X.2021.1902821 (PMC7993373; doi:10.1080/0886022X.2021.1902821)
Supplement: Supplemental Material [file IRNF_A_1902821_SM2584.pdf]

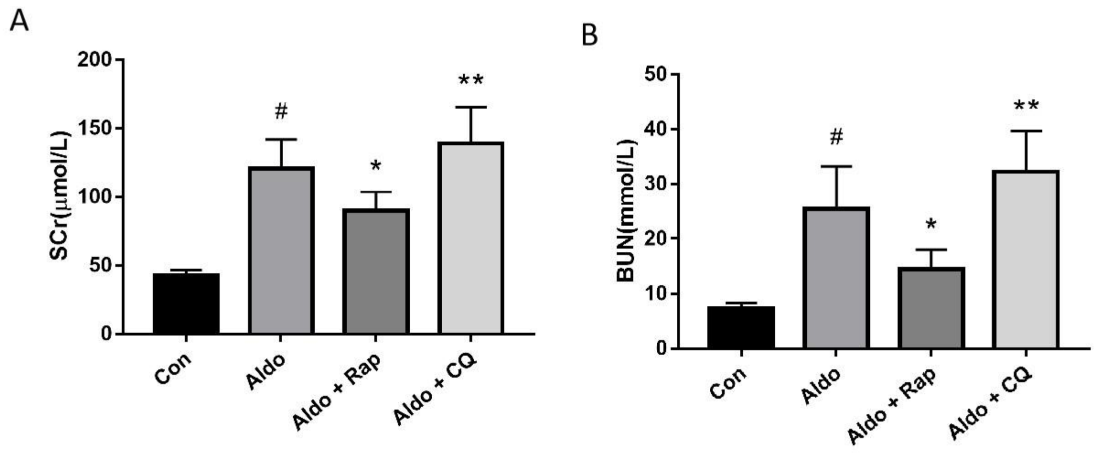

Supplementary figure1: The levels of SCr and BUN

(A) The levels of SCr (n=6). $^{\#}P<0.05$ vs. normal control, $^{*}P<0.05$ vs. Aldo alone. $^{**}P=0.106$. (B) The levels of BUN (n=6). $^{\#}P<0.05$ vs. normal control, $^{*}P<0.05$ vs. Aldo alone.$^{**}P=0.056$.

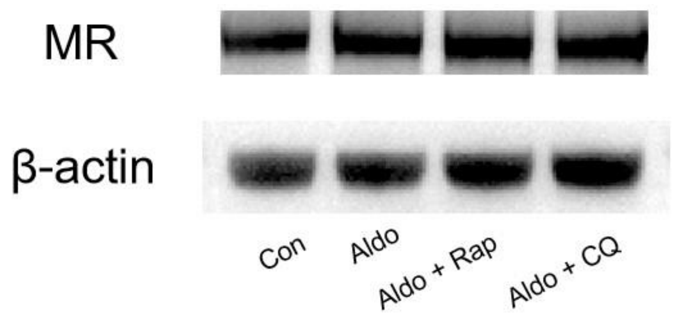

Supplementary figure2: The expression of mineralocorticoid receptor

(A) Western blot analysis revealed the expression of mineralocorticoid receptor in kidney after various treatments as indicated.
